# Supplementary figures and images for: Selection and evaluation of lactic acid bacteria from chicken feces in Thailand as potential probiotics
Source: PeerJ. 2023 Dec 14;11:e16637. doi: 10.7717/peerj.16637 (PMC10725671; doi:10.7717/peerj.16637)

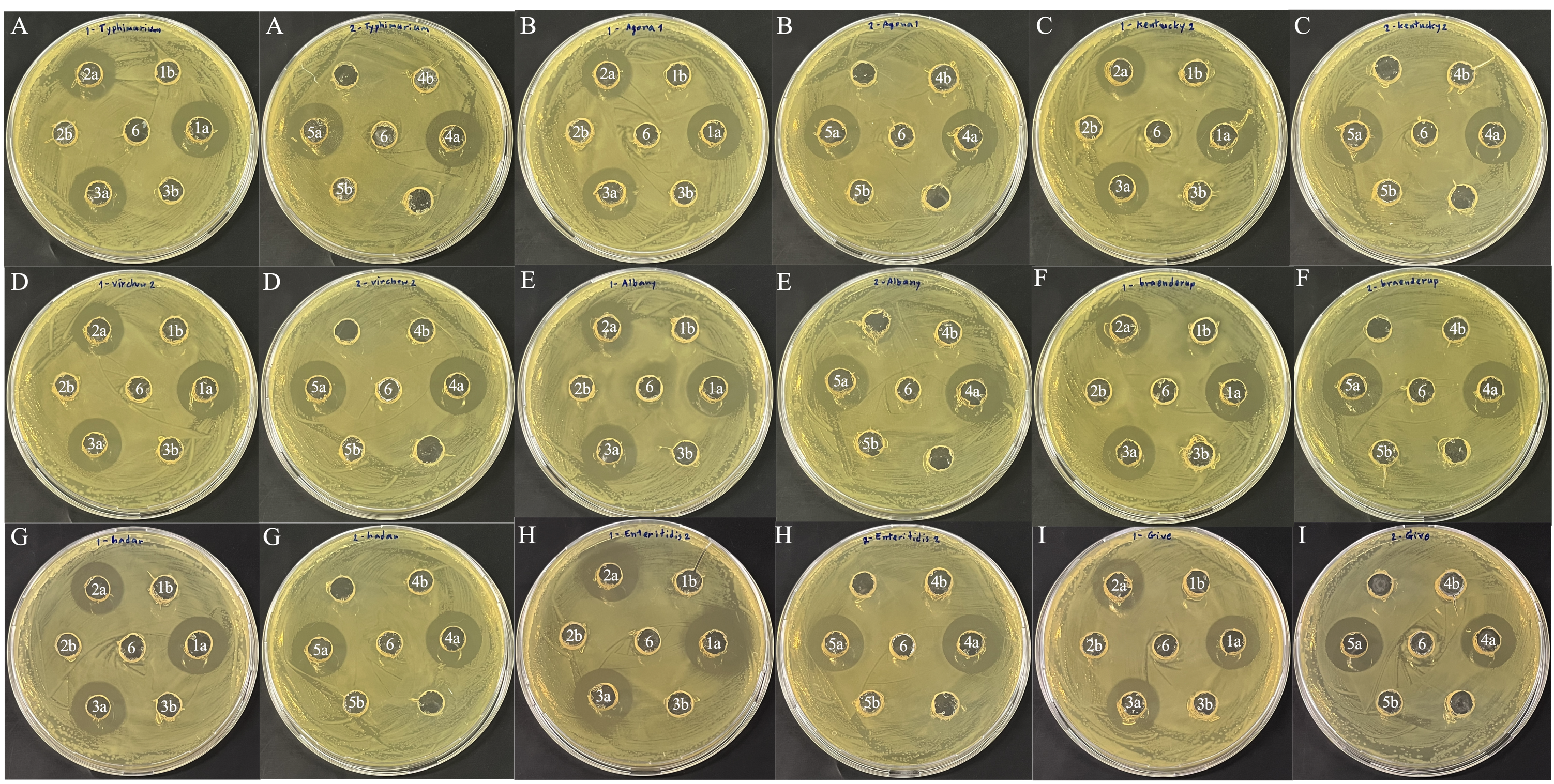

Supplement: Supplemental Information 6 — S1: Antimicrobial activity of CFS (a) and NCFS (b) from 5 selected LAB isolates including; L. salivarius BF12 (1); P. acidilactici BF9 (2); P. acidilactici BF14 (3); P. acidilactici BYF20 (4); P. acidilactici BYF26 (5) against some isolates of 9 serovars of Salmonella enterica including; S. Typhimurium 3 (A); S. Agona 1 (B); S. Kentucky 2 (C); S. Virchow 2 (D); S. Albany (E); S. Braenderup (F); S. Hadar (G); S. Enteritidis 2 (H); S. Give (I). [file peerj-11-16637-s006.png]
