# Supplementary material for: Selection and evaluation of lactic acid bacteria from chicken feces in Thailand as potential probiotics
Source: PeerJ. 2023 Dec 14;11:e16637. doi: 10.7717/peerj.16637 (PMC10725671; doi:10.7717/peerj.16637)
Supplement: Supplemental Information 8 — Use of the real-time PCR assay only identified ermB, an erythromycin resistance gene, and only in P. acidilactici isolates BF9 and BF14. [file peerj-11-16637-s008.pdf]

ร้านฉลาดคิด® by Siriraj
งานพัฒนาและสร้างประโยชน์จากนวัตกรรมและเทคโนโลยี
ฝ่ายวิจัย สำนักงานคณบดี คณะแพทยศาสตร์ศิริราชพยาบาล
มหาวิทยาลัยมหิดล โทร.0-24192995-7 โทรสาร.0-24183307
เลขประจำตัวผู้เสียภาษี 0994000158378

Room 542, Research Laboratory Unit, Department of Microbiology, Faculty of Medicine Siriraj Hospital, Mahidol University, 2 Wanglang Rd., Siriraj, Bangkoknoi, Bangkok 10700, Thailand

Antimicrobial Resistant Gene Detection Report

Date :05.11.2020

Name & Address of customer		Veterinary Microbiology 12th Floor, 60th year Veterinary Science building Faculty of Veterinary Science, Chulalongkorn University, Henri-Dunant Rd., Pathumwan, Bangkok 10330	
Sample ID / type		Ped. BYF 20	PTA220
Date of sample receipt	27-10-2020	Method of test	Real-time PCR with specific probes
Date of sample testing	29-10-2020 to 04-11-2020	Condition of sample when receive	Genomic DNA

Results

	Antimicrobial classes	Target gene	Results
1	β-lactams (penicillin, amoxicillin, cephalosporin)	CTX-M1	NF
2		CTX-M2-M74	NF
3		CTX-M8-M25	NF
4		CTX-M9	NF
5		PER	NF
6		VEB	NF
7		CMY1-MOX	NF
8		CMY2-LAT	NF
9		DHA	NF
10		FOX	NF
11		ACT-MIR	NF
12		OXA-1	NF
15	Carbapenems	KPC	NF
16		NDM	NF
17		VIM	NF
18		IMP	NF
19		OXA-48	NF
20	Folate pathway inhibitors	*sul1*	NF
21		*sul2*	NF
22		*sul3*	NF
23		*dfrA1*	NF
24		*dfrA5-14*	NF
25		*dfrA12*	NF
26		*dfrA17*	NF

	Antimicrobial classes	Target gene	Results
27	Polymyxins	*mcr-1*	NF
28		*mcr-2*	NF
29	Tetracyclines	*tetA*	NF
30		*tetB*	NF
31	Phenicols	*cmlA*	NF
32		*floR*	NF
33		*catA1*	NF
34		*catB3*	NF
35	Aminoglycosides	*aacC1*	NF
36		*aacC2*	NF
37		*aacC4*	NF
38		*aphA1*	NF
39		*aadA1-2-17*	NF
40		*aadB*	NF
41		*armA*	NF
42		*rmtB*	NF
43	Macrolides	*ermB*	NF
44		*mphA*	NF
45	Quinolones	*qnrA*	NF
46		*qnrS*	NF
47		*qnrB1*	NF
48		*qnrB4*	NF
49		*QepA*	NF

~ 3-8 X10^6 cells was tested. Positive grading criteria: 1+: ≥ 10^1-10^2, 2+: >10^2-10^3 and 3+ = >10^3 positive cells NF = Not found

Tested person: *Rattapha Chinli*
(Ms. Rattapha Chinli)

Authorized person: *Suporn Foongladda*
(Assoc.Prof.Dr. Suporn Foongladda)

Tel: 02-4199811, 0819390258 e-mail: suporn.foo@mahidol.ac.th

ร้านฉลาดคิด® by Siriraj
งานพัฒนาและสร้างประโยชน์จากนวัตกรรมและเทคโนโลยี
ฝ่ายวิจัย สำนักงานคณบดี คณะแพทยศาสตร์ศิริราชพยาบาล
มหาวิทยาลัยมหิดล โทร.0-24192995-7 โทรสาร.0-24183307
เลขประจำตัวผู้เสียภาษี 0994000158378

Room 542, Research Laboratory Unit, Department of Microbiology, Faculty of Medicine Siriraj Hospital, Mahidol University, 2 Wanglang Rd., Siriraj, Bangkoknoi, Bangkok 10700, Thailand

Antimicrobial Resistant Gene Detection Report

Date :05.11.2020

Name & Address of customer		Veterinary Microbiology 12th Floor, 60th year Veterinary Science building Faculty of Veterinary Science, Chulalongkorn University, Henri-Dunant Rd., Pathumwan, Bangkok 10330	
Sample ID / type		Ped. BYF 26	PTA221
Date of sample receipt	27-10-2020	Method of test	Real-time PCR with specific probes
Date of sample testing	29-10-2020 to 04-11-2020	Condition of sample when receive	Genomic DNA

Results

	Antimicrobial classes	Target gene	Results
1	β-lactams (penicillin, amoxicillin, cephalosporin)	CTX-M1	NF
2		CTX-M2-M74	NF
3		CTX-M8-M25	NF
4		CTX-M9	NF
5		PER	NF
6		VEB	NF
7		CMY1-MOX	NF
8		CMY2-LAT	NF
9		DHA	NF
10		FOX	NF
11		ACT-MIR	NF
12		OXA-1	NF
15	Carbapenems	KPC	NF
16		NDM	NF
17		VIM	NF
18		IMP	NF
19		OXA-48	NF
20	Folate pathway inhibitors	*sul1*	NF
21		*sul2*	NF
22		*sul3*	NF
23		*dfrA1*	NF
24		*dfrA5-14*	NF
25		*dfrA12*	NF
26		*dfrA17*	NF

	Antimicrobial classes	Target gene	Results
27	Polymyxins	*mcr-1*	NF
28		*mcr-2*	NF
29	Tetracyclines	*tetA*	NF
30		*tetB*	NF
31	Phenicols	*cmlA*	NF
32		*floR*	NF
33		*catA1*	NF
34		*catB3*	NF
35	Aminoglycosides	*aacC1*	NF
36		*aacC2*	NF
37		*aacC4*	NF
38		*aphA1*	NF
39		*aadA1-2-17*	NF
40		*aadB*	NF
41		*armA*	NF
42		*rmtB*	NF
43	Macrolides	*ermB*	NF
44		*mphA*	NF
45	Quinolones	*qnrA*	NF
46		*qnrS*	NF
47		*qnrB1*	NF
48		*qnrB4*	NF
49		*QepA*	NF

~ 3-8 X10^6 cells was tested. Positive grading criteria: 1+: ≥ 10^1-10^2, 2+: >10^2-10^3 and 3+ = >10^3 positive cells NF = Not found

Tested person: Rattapha Chinli
(Ms. Rattapha Chinli)

Authorized person: Suporn Foongladda
(Assoc.Prof.Dr. Suporn Foongladda)

Tel: 02-4199811, 0819390258 e-mail: suporn.foo@mahidol.ac.th

ร้านฉลาดคิด® by Siriraj
งานพัฒนาและสร้างประโยชน์จากนวัตกรรมและเทคโนโลยี
ฝ่ายวิจัย สำนักงานคณบดี คณะแพทยศาสตร์ศิริราชพยาบาล
มหาวิทยาลัยมหิดล โทร.0-24192995-7 โทรสาร.0-24183307
เลขประจำตัวผู้เสียภาษี 0994000158378

Room 542, Research Laboratory Unit, Department of Microbiology, Faculty of Medicine Siriraj Hospital, Mahidol University, 2 Wanglang Rd., Siriraj, Bangkoknoi, Bangkok 10700, Thailand

Antimicrobial Resistant Gene Detection Report

Date :05.11.2020

Name &<br>Address of customer		Veterinary Microbiology<br>12th Floor, 60th year Veterinary Science building Faculty of Veterinary Science, Chulalongkorn University, Henri-Dunant Rd., Pathumwan, Bangkok 10330	
Sample ID / type		Ped. BF 9	PTA222
Date of sample receipt	27-10-2020	Method of test	Real-time PCR with specific probes
Date of sample testing	29-10-2020 to 04-11-2020	Condition of sample when receive	Genomic DNA

Results

	Antimicrobial classes	Target gene	Results
1	β-lactams	CTX-M1	NF
2	(penicillin,	CTX-M2-M74	NF
3	amoxicillin,	CTX-M8-M25	NF
4	cephalosporin)	CTX-M9	NF
5		PER	NF
6		VEB	NF
7		CMY1-MOX	NF
8		CMY2-LAT	NF
9		DHA	NF
10		FOX	NF
11		ACT-MIR	NF
12		OXA-1	NF
15	Carbapenems	KPC	NF
16		NDM	NF
17		VIM	NF
18		IMP	NF
19		OXA-48	NF
20	Folate pathway	*sul1*	NF
21	inhibitors	*sul2*	NF
22		*sul3*	NF
23		*dfrA1*	NF
24		*dfrA5-14*	NF
25		*dfrA12*	NF
26		*dfrA17*	NF

	Antimicrobial classes	Target gene	Results
27	Polymyxins	*mcr-1*	NF
28		*mcr-2*	NF
29	Tetracyclines	*tetA*	NF
30		*tetB*	NF
31	Phenicols	*cmlA*	NF
32		*floR*	NF
33		*catA1*	NF
34		*catB3*	NF
35	Aminoglycosides	*aacC1*	NF
36		*aacC2*	NF
37		*aacC4*	NF
38		*aphA1*	NF
39		*aadA1-2-17*	NF
40		*aadB*	NF
41		*armA*	NF
42		*rmtB*	NF
43	Macrolides	*ermB*	3+
44		*mphA*	NF
45	Quinolones	*qnrA*	NF
46		*qnrS*	NF
47		*qnrB1*	NF
48		*qnrB4*	NF
49		*QepA*	NF

~ 3-8 X10^6 cells was tested. Positive grading criteria: 1+: ≥ 10^1-10^2, 2+: >10^2-10^3 and 3+ = >10^3 positive cells NF = Not found

Tested person: *Rattapha Chinli*
(Ms. Rattapha Chinli)

Authorized person: *Suporn Foongladda*
(Assoc.Prof.Dr. Suporn Foongladda)

Tel: 02-4199811, 0819390258 e-mail: suporn.foo@mahidol.ac.th

ร้านฉลาดคิด® by Siriraj
งานพัฒนาและสร้างประโยชน์จากนวัตกรรมและเทคโนโลยี
ฝ่ายวิจัย สำนักงานคณบดี คณะแพทยศาสตร์ศิริราชพยาบาล
มหาวิทยาลัยมหิดล โทร.0-24192995-7 โทรสาร.0-24183307
เลขประจำตัวผู้เสียภาษี 0994000158378

Room 542, Research Laboratory Unit, Department of Microbiology, Faculty of Medicine Siriraj Hospital, Mahidol University, 2 Wanglang Rd., Siriraj, Bangkoknoi, Bangkok 10700, Thailand

Antimicrobial Resistant Gene Detection Report

Date :05.11.2020

Name & Address of customer		Veterinary Microbiology<br>12th Floor, 60th year Veterinary Science building Faculty of Veterinary Science, Chulalongkorn University, Henri-Dunant Rd., Pathumwan, Bangkok 10330	
Sample ID / type		Ped. BF 14	PTA223
Date of sample receipt	27-10-2020	Method of test	Real-time PCR with specific probes
Date of sample testing	29-10-2020 to 04-11-2020	Condition of sample when receive	Genomic DNA

Results

	Antimicrobial classes	Target gene	Results
1	β-lactams (penicillin, amoxicillin, cephalosporin)	CTX-M1	NF
2		CTX-M2-M74	NF
3		CTX-M8-M25	NF
4		CTX-M9	NF
5		PER	NF
6		VEB	NF
7		CMY1-MOX	NF
8		CMY2-LAT	NF
9		DHA	NF
10		FOX	NF
11		ACT-MIR	NF
12		OXA-1	NF
15	Carbapenems	KPC	NF
16		NDM	NF
17		VIM	NF
18		IMP	NF
19		OXA-48	NF
20	Folate pathway inhibitors	*sul1*	NF
21		*sul2*	NF
22		*sul3*	NF
23		*dfrA1*	NF
24		*dfrA5-14*	NF
25		*dfrA12*	NF
26		*dfrA17*	NF

	Antimicrobial classes	Target gene	Results
27	Polymyxins	*mcr-1*	NF
28		*mcr-2*	NF
29	Tetracyclines	*tetA*	NF
30		*tetB*	NF
31	Phenicols	*cmlA*	NF
32		*floR*	NF
33		*catA1*	NF
34		*catB3*	NF
35	Aminoglycosides	*aacC1*	NF
36		*aacC2*	NF
37		*aacC4*	NF
38		*aphA1*	NF
39		*aadA1-2-17*	NF
40		*aadB*	NF
41		*armA*	NF
42		*rmtB*	NF
43	Macrolides	*ermB*	3+
44		*mphA*	NF
45	Quinolones	*qnrA*	NF
46		*qnrS*	NF
47		*qnrB1*	NF
48		*qnrB4*	NF
49		*QepA*	NF

~ 3-8 X10^6 cells was tested. Positive grading criteria: 1+: ≥ 10^1-10^2, 2+: >10^2-10^3 and 3+ = >10^3 positive cells NF = Not found

Tested person: Rattapha Chinli (Ms. Rattapha Chinli)

Authorized person: Suporn Foongladda (Assoc.Prof.Dr. Suporn Foongladda)

Tel: 02-4199811, 0819390258 e-mail: suporn.foo@mahidol.ac.th

ร้านฉลาดคิด® by Siriraj
งานพัฒนาและสร้างประโยชน์จากนวัตกรรมและเทคโนโลยี
ฝ่ายวิจัย สำนักงานคณบดี คณะแพทยศาสตร์ศิริราชพยาบาล
มหาวิทยาลัยมหิดล โทร.0-24192995-7 โทรสาร.0-24183307
เลขประจำตัวผู้เสียภาษี 0994000158378

Room 542, Research Laboratory Unit, Department of Microbiology, Faculty of Medicine Siriraj Hospital, Mahidol University, 2 Wanglang Rd., Siriraj, Bangkoknoi, Bangkok 10700, Thailand

Antimicrobial Resistant Gene Detection Report

Date :05.11.2020

Name & Address of customer		Veterinary Microbiology<br>12th Floor, 60th year Veterinary Science building Faculty of Veterinary Science, Chulalongkorn University, Henri-Dunant Rd., Pathumwan, Bangkok 10330	
Sample ID / type		Lac. BF 12	PTA224
Date of sample receipt	27-10-2020	Method of test	Real-time PCR with specific probes
Date of sample testing	29-10-2020 to 04-11-2020	Condition of sample when receive	Genomic DNA

Results

	Antimicrobial classes	Target gene	Results
1	β-lactams (penicillin, amoxicillin, cephalosporin)	CTX-M1	NF
2		CTX-M2-M74	NF
3		CTX-M8-M25	NF
4		CTX-M9	NF
5		PER	NF
6		VEB	NF
7		CMY1-MOX	NF
8		CMY2-LAT	NF
9		DHA	NF
10		FOX	NF
11		ACT-MIR	NF
12		OXA-1	NF
15	Carbapenems	KPC	NF
16		NDM	NF
17		VIM	NF
18		IMP	NF
19		OXA-48	NF
20	Folate pathway inhibitors	*sul1*	NF
21		*sul2*	NF
22		*sul3*	NF
23		*dfrA1*	NF
24		*dfrA5-14*	NF
25		*dfrA12*	NF
26		*dfrA17*	NF

	Antimicrobial classes	Target gene	Results
27	Polymyxins	*mcr-1*	NF
28		*mcr-2*	NF
29	Tetracyclines	*tetA*	NF
30		*tetB*	NF
31	Phenicols	*cmlA*	NF
32		*floR*	NF
33		*catA1*	NF
34		*catB3*	NF
35	Aminoglycosides	*aacC1*	NF
36		*aacC2*	NF
37		*aacC4*	NF
38		*aphA1*	NF
39		*aadA1-2-17*	NF
40		*aadB*	NF
41		*armA*	NF
42		*rmtB*	NF
43	Macrolides	*ermB*	NF
44		*mphA*	NF
45	Quinolones	*qnrA*	NF
46		*qnrS*	NF
47		*qnrB1*	NF
48		*qnrB4*	NF
49		*QepA*	NF

~ 3-8 X10^6 cells was tested. Positive grading criteria: 1+: ≥ 10^1-10^2, 2+: >10^2-10^3 and 3+ = >10^3 positive cells NF = Not found

Tested person: Rattapha Chinli
(Ms. Rattapha Chinli)

Authorized person: Suporn Foongladda
(Assoc.Prof.Dr. Suporn Foongladda)

Tel: 02-4199811, 0819390258 e-mail: suporn.foo@mahidol.ac.th
